# Pretransplant active disease status and HLA class II mismatching are associated with increased incidence and severity of cytokine release syndrome after haploidentical transplantation with posttransplant cyclophosphamide

**DOI:** 10.1002/cam4.2607

**Published:** 2019-11-08

**Authors:** Jacopo Mariotti, Daniela Taurino, Fabrizio Marino, Stefania Bramanti, Barbara Sarina, Lucio Morabito, Chiara De Philippis, Clara Di Vito, Domenico Mavilio, Carmelo Carlo‐Stella, Matteo Della Porta, Armando Santoro, Luca Castagna

**Affiliations:** ^1^ Bone Marrow Transplant Unit Humanitas Clinical and Research Center-IRCCS-via Manzoni 56 Rozzano Italy; ^2^ Unit of Clinical and Experimental Immunology Humanitas Clinical and Research Center Rozzano Italy; ^3^ Department of Medical Biotechnology and Translational Medicine University of Milano Milano Italy; ^4^ Humanitas Clinical and Research Hospital Humanitas University Rozzano Italy

**Keywords:** cytokine release syndrome, disease burden, haploidentical stem cell transplant with posttransplant cyclophosphamide, HLA mismatch

## Abstract

Cytokine release syndrome (CRS) represents a life‐threatening side effect after haploidentical stem cell transplantation (Haplo‐SCT) with posttransplant cyclophosphamide (PT‐Cy). Factors predictive of CRS development is still a matter of debate. We retrospectively analyzed 102 consecutive patients receiving a bone marrow (BM) (n = 42) or peripheral blood stem cells (PBSC) (n = 60) Haplo‐SCT with PT‐Cy. The two cohorts were similar in main patients’ characteristics besides disease type (*P* = .02). Cumulative incidence of grades 1, 2, and ≥3 CRS was 80%, 52%, and 15% at a median of 2, 4, and 7 days, respectively. Moderate/High‐grade fever (39°‐41°), grade 1 and grade ≥3 CRS occurred more frequently after PBSC relative to BM grafts (68% vs 33%, *P* = .0005; 87% vs 71%, *P* = .009; 20% vs 7%, *P* = .07). Only patients experiencing grade ≥3 CRS had a worse outcome in terms of 1‐year overall survival (OS) and nonrelapse mortality (NRM): 39% vs 80% (*P* = .002) and 40% vs 8% (*P* = .005), respectively. By univariate analysis the only factors associated with the increased risk of ≥3 CRS were pretransplant disease status (8% for complete remission, 11% for partial remission, and 38% for active disease, *P* = .002), HLA‐DRB1 mismatching (57% vs 14%, *P* = .007), and PBSC graft (*P* = .07). By multivariable analysis, only pretransplant disease status (hazard ratio, HR: 6.84, *P* = .005) and HLA‐DRB1 mismatching (HR: 17.19, *P* = .003) remained independent predictors of grade ≥3 CRS. Only grade ≥3 CRS is clinically relevant for the final outcome of patients receiving Haplo‐SCT with PT‐Cy, is more frequent after a PBSC graft and is associated with pretransplant active disease and HLA‐DRB1 mismatching.

## Graphical abstract

Severe cytokine release syndrome (CRS) may occur after T‐cell‐replete haploidentical stem cell transplantation and conditions nonrelapse mortality. Disease burden and HLA mismatch in the graft‐vs‐host‐disease direction are the main factors affecting the occurrence of severe CRS.

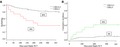

## 1 INTRODUCTION

T‐cell‐replete haploidentical stem cell transplantation (Haplo‐SCT) with high‐dose posttransplant cyclophosphamide (PT‐Cy) represents an emerging alternative option for patients with hematologic malignancies when a HLA identical sibling or a matched related donor is not available.^1^, ^2^ Bone marrow (BM) stem cells represented the first type of graft used for Haplo‐SCT,^3^ but peripheral blood stem cells (PBSC) are more often employed because of the ease and convenience of collection.^2^

Cytokine release syndrome (CRS) is a potentially life‐threatening toxic effect of immunotherapy and was first described after treatment with blinatumomab^4^ or with chimeric antigen receptor (CAR)‐modified T cells.^5^, ^6^ CRS results from the activation and cytotoxic‐mediated damage of monocytes, macrophages, and different lymphocyte populations with a massive release of inflammatory cytokines, including IL‐6, IFNγ, and IL‐2. CRS occurs as a systemic inflammation with a variety of symptoms ranging from nonspecific and not life‐threatening symptoms, such as fever, rigors, malaise, headaches, myalgias, arthralgias, vascular leak, hypotension, to more severe organ dysfunction like respiratory and renal insufficiency until multisystem organ failure.

More recently, a haplo‐immuno storm similar to CRS occurring within the first 14 days after PBSC Haplo‐SCT with PT‐Cy was described by Abboud et al.^7^ Severe CRS was associated with poor clinical outcomes, including nonrelapse mortality (NRM), overall survival (OS), and delayed neutrophil and platelet engraftment. While Abboud et al^7^ could not identify any predictive factors associated with the increased risk of CRS, Raj et al^8^ described a higher incidence of grade ≥2 CRS after PBSC relative to BM grafts. Fever is the most common manifestation of CRS and several reports have consistently described the occurrence of a noninfectious fever within the first 5 days after Haplo‐SCT.^9^, ^10^, ^11^, ^12^, ^13^, ^14^ This process was firstly called infusion‐related febrile reaction and was found to be associated with a higher risk of engraftment syndrome and acute graft‐vs‐host‐disease (aGVHD).^12^, ^13^ Early post‐SCT fever correlated with the dose of CD34^+^ cells content of the graft^12^, ^13^ and more recently with the HLA II mismatching and myeloablative conditioning regimen.^15^

Here we report a retrospective study performed at our institution with the aim of investigating the outcome and risk factors of patients with CRS after Haplo‐SCT with PT‐Cy. We first hypothesize that patients receiving PBSC grafts were at higher risk of developing severe CRS relative to BM grafts and we aimed at comparing these two cohorts. We also analyze whether other variables, including HLA mismatching, may correlate with an increased risk of CRS.

## 2 PATIENTS AND METHODS

This is a retrospective study comprising 102 consecutive patients with hematologic malignancies receiving T‐cell‐replete Haplo‐SCT with PT‐Cy at a single institution between January 2014 and December 2017. The study was approved from the Institutional Review Board at our institution and patients provided informed consent for the retrospective collection of their data. All procedures followed were in accordance with the ethical standards of the responsible committee on human experimentation (institutional and national) and with the Helsinki Declaration of 1975, as revised in 2008. Patients were ineligible for allogeneic transplant if they had active uncontrolled infections, a CNS disease, a Karnofsky performance status <60 or severe organ dysfunction, including a left ventricular ejection fraction <40%, DLCO <50% or creatinine clearance <50 mL/min. The comorbidity index of each patient was calculated using the hematopoietic cell transplant‐comorbidity index (HCT‐CI) system.^16^

### 2.1 Conditioning regimen and GVHD prophylaxis

Three different conditioning regimens prior to Haplo‐SCT were employed: (a) Cy 14.5 mg/kg on days −6 and −5, fludarabine 30 mg/m^2^ from day −6 to day −2 and low‐dose TBI (2 Gy) on day −1; (b) thiotepa 10 mg/kg on day −5, cyclophosphamide 30 mg/kg on day −4 and −3, fludarabine 30 mg/m^2^ on day −4 and −3; (c) thiotepa 5 mg/kg on day‐5, fludarabine 50 mg/m^2^ from day −4 to day −2, busulphan iv 3.2 mg/kg on day −4 and −3. GVHD prophylaxis consisted of posttransplantation Cy 50 mg/kg (PT‐Cy) administered on day +3 and +4, tacrolimus or cyclosporine A and mycophenolate mofetil as previously described.^17^ Granulocyte colony‐stimulating factor (G‐CFS) was started on day +5 in all patients as previously described.^17^

### 2.2 Stem cell sources and donors

Potential family members were typed at the HLA‐A, HLA‐B, and HLA‐DRB1 loci at high level of resolution. Selected donors were also typed at the HLA‐C locus at a high‐resolution level. Some donors underwent BM harvest under general anesthesia for a target dose of 3‐4 × 10^8^ nuclear cells/kg of recipient weight. Other donors were mobilized by the subcutaneous administration of G‐CFS for 5‐6 days at 10 μg/kg/day. The target was a minimum of 4 × 10^6^ CD34 cells/kg. Unmanipulated BM and PBSC were used for stem cell support on day 0. The choice of type of graft was related to physician or donor preference, therefore the was no particular bias due to graft type.

### 2.3 Supportive care, engraftment, and GVHD evaluation

Supportive care was provided as described previously.^17^ Neutrophil engraftment was defined as the first of 3 consecutive days with an absolute neutrophil count of 0.5 × 10^9^/L after transplantation. Platelet engraftment was defined as a platelet count of 20 × 10^9^/L, with no transfusions during the preceding 7 days. aGVHD was graded according to the Keystone criteria,^18^ and chronic GVHD (cGVHD) following the National Institutes of Health criteria.^19^

### 2.4 CRS definition

CRS was graded according to the original criteria from Lee et al^20^ and the recent update by Abboud et al^7^ in order to comprise also altered mental status and new onset cardiomyopathy (Table S1). Symptoms occurring before day +14 were included as in the manuscript by Abboud et al^7^ and Raj et al.^8^ The detailed clinical data from posttransplantation days 0‐14 were collected and used to grade CRS. These data included fever curves, vital signs, renal and hepatic function tests, CRP levels, the development of vasopressor dependence, oxygen requirement, and the need for mechanical ventilation. Pre‐ and posttransplantation ejection fractions from echocardiogram and or nuclear perfusion study results were also collected. Patients with a documented infection did not receive a CRS grade. CRS was distinguished from engraftment syndrome, which can also present as noninfectious fever and capillary leak, by onset before day +14 and the absence of skin rash.

### 2.5 Statistical analysis

Categorical variables are expressed as number and proportion, and continuous variables are expressed as median and range. For the calculation of CRS, documented infections were considered as competing event. For the calculation of NRM, disease relapse or progression was treated as a competing event, whereas NRM was the competing event for the calculation of cumulative incidence of relapse or progression.^21^ The cumulative incidence of aGVHD was estimated considering death not related to aGVHD within a year posttransplant as a competing event. cGVHD was estimated only for patients alive at day +100, considering death not related to cGVHD within 2 years posttransplant as a competing event. The Kaplan‐Meier method was used for the OS analysis.^22^ Comparisons between groups were made using log‐rank and Gray tests when indicated. Cox regression (Cox) was performed to identify any significant association between the main risk factors and the outcomes of interest^23^; variables with a *P* < .20 entered in the multivariable analysis and only those with a *P* ≤ .05 were retained in the final model. SPSS version 19.0 (IBM) and EZR (“Easy R”; R Institute for Statistical Computing) were used.

## 3 RESULTS

From February 2014 to December 2017, 102 consecutive patients received a Haplo‐SCT either from a BM (n = 42) or PBSC (n = 60) graft source. Median follow‐up time for alive patients was 26.8 months (range 5‐58). Patients’ characteristics are summarized in Table 1. The two cohorts were similar in terms of median donor and recipient age, pretransplant disease status, CMV serostatus, HCT‐CI, conditioning regimen, donor/recipient relationship, and sex mismatch. The only difference was represented by disease type with acute myeloid leukemia/myelodysplastic syndrome as the most frequent malignancy among PBSC graft recipient (50%) and Hodgkin (HL: 36%) and non‐Hodgkin (NHL: 29%) lymphomas as the most frequent malignancy in the BM cohort (*P* = .02).

**Table 1:** Characteristics of patients receiving haploidentical‐SCT from a BM or PBSC graft

Characteristics	Total	PBSC graft	BM graft	*P*
No. patients	102	60	42	
Median recipient age	52 (20‐72)	56 (20‐72)	48 (22‐70)	.08
Median donor age	41 (18‐71)	41 (18‐71)	38 (20‐67)	.78
Gender M/F	46/56	35/25	21/21	.42
Disease type				**.02**
AML/MDS	39 (38%)	30 (50%)	9 (21%)	
ALL	6 (6%)	3 (5%)	3 (7%)	
Myeloproliferative	2 (2%)	2 (3%)	0	
HL	30 (30%)	16 (27%)	15 (36%)	
NHL	19 (19%)	7 (12%)	12 (29%)	
Other lymphoproliferative	5 (5%)	2 (3%)	3 (7%)	
Disease status pre‐Allo				.18
CR	63 (62%)	35 (58%)	28 (67%)	
PR	18 (17%)	9 (15%)	9 (21%)	
SD/PD	21 (21%)	16 (27%)	5 (12%)	
Conditioning regimens				.61
Nonmyeloablative	35 (34%)	23 (38%)	12 (29%)	
Reduced intensity	53 (52%)	29 (48%)	24 (57%)	
Myeloablative	14 (14%)	8 (14%)	6 (14%)	
HCT‐CI	N = 100	N = 59	N = 41	.68
0‐2	53 (53%)	30 (51%)	23 (56%)	
≥3	47 (47%)	29 (49%)	18 (44%)	
CMV serostatus	N = 100	N = 59	N = 41	98
Neg/Neg	4 (4%)	2 (4%)	2 (5%)	
Pos/Neg	15 (15%)	9 (15%)	6 (15%)	
Pos/Pos	64 (64%)	38 (64%)	26 (63%)	
Neg/Pos	17 (17%)	10 (17%)	7 (17%)	
Gender mismatch				.08
Female→male	21 (21%)	16 (26%)	5 (12%)	
Others	81 (81%)	44 (74%)	37 (88%)	
Donor type				.62
Child	40 (39%)	25 (42%)	15 (36%)	
Sibling	45 (45%)	27 (46%)	18 (43%)	
Parent	14 (13%)	6 (9%)	8 (19%)	
Cousin/nephew	3 (3%)	2 (3%)	1 (2%)	

Values are bold when *P* is statisctically significant (*P* < .05).

Abbreviations: ALL, acute lymphoblastic leukemia; AML, acute myeloid leukemia; BM, bone marrow; CMV, cytomegalovirus; CR, complete remission; HCT‐CI, hematopoietic cell transplant‐comorbidity index; HL, Hodgkin lymphoma; M/F, male/female; MDS, Myelodysplastic syndrome; NHL, non‐Hodgkin lymphoma; PBSC, peripheral blood stem cell; PD, progressive disease; PMF, primary myelofibrosis; PR, partial remission; SCT, stem cell transplantation; SD, stable disease.

### 3.1 Incidence and characteristics of CRS

**Figure 1:**
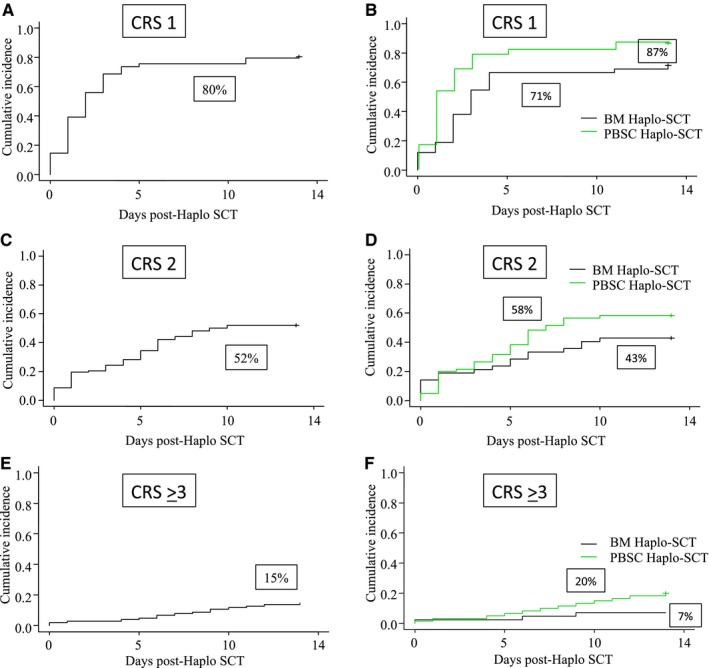
Cumulative incidence of CRS after Haplo‐SCT with PT‐Cy: (A) Cumulative incidence of grade 1 CRS in the whole population; (B) after PBSC or BM grafts; (C) Cumulative incidence of grade 2 CRS in the whole population; (D) after PBSC or BM grafts; (E) Cumulative incidence of grade ≥3 CRS in the whole population; and (F) after PBSC or BM grafts. BM, bone marrow; CRS, cytokine release syndrome; Haplo‐SCT, haploidentical stem cell transplantation; PBSC, peripheral blood stem cells; PT‐Cy, posttransplant cyclophosphamide

Eighty‐two patients experienced grade 1 CRS at a median of 2 days (range 0‐14) post‐SCT, 53 had grade 2 CRS at a median of 4 days (range 0‐10), and 15 had grade ≥3 CRS at a median of 7 days (range 0‐14) post‐SCT (Table 2). Cumulative incidence of grades 1, 2, and ≥3 CRS was 80% (95% confidential interval [CI]: 71‐87), 52% (95% CI: 42‐61), and 15% (95% CI: 9‐22), respectively (Figure 1A, C, and E). Cumulative incidence of grades 1 and 3 CRS was higher after PBSC relative to BM grafts recipients (87% vs 71%, *P* = .009 and 20% vs 7%, *P* = .07), while there was no difference in terms of grade 2 CRS between the two stem cell sources (58% vs 43%, *P* = .15) (Figure 1B, D, and F).

**Table 2:** CRS grading in PBSC and BM Haplo‐SCT

Days 0‐14	PBSC (n = 60)	BM (n = 42)	*P*
All grades	55 (92%)	33 (79%)	.08
Grade 1	52 (87%)	30 (71%)	.07
Grade 2	35 (58%)	18 (43%)	.15
Grade 3‐4	12 (20%)	3 (7%)	.09

Abbreviations: BM, bone marrow; CRS, cytokine release syndrome; PBSC, peripheral blood stem cell; Haplo‐SCT, haploidentical stem cell transplantation.

Signs and symptoms of CRS are summarized in Table 3. Thirteen patients had positive blood cultures before any sign of CRS and were considered as competing event for the analysis. Seven patients had no fever. Fever was the most frequent event (82 patients, grade 1 CRS) with more patients experiencing low‐grade fever (<38°C) after BM Haplo‐SCT, and more patients with intermediate (38‐39°C) and high‐grade fever (>40°C) after PBSC transplant (*P* = .0005). Hypotension and liver toxicity were the other more frequent manifestations of CRS with no difference between PBSC and BM grafts. Thirteen patients required oxygen supply and 15 had altered mental status: both side effects were more frequent after PBSC Haplo‐SCT. In particular, only PBSC recipients required higher volumes of oxygen supply (>3 L/min) relative to BM transplants (5 vs 0).

**Table 3:** Characteristics of CRS in BM or PBSC Haplo‐SCT

Characteristics	All	PBSC (n = 60)	BM (n = 42)	*P*
No fever	7	3 (5%)	4 (10%)	**.0005**
Fever				
38‐39	31	11 (19%)	20 (48%)	
39‐40	30	24 (40%)	6 (13%)	
>40	21	17 (28%)	4 (10%)	
Positive blood cultures	13	5 (8%)	8 (19%)	
Hypotension (requiring fluids)				.17
None	65	34 (56%)	31 (74%)	
Low dose pressure	35	25 (42%)	10 (24%)	
High dose pressures	2	1 (2%)	1 (2%)	
O_2_ requirement		8 (13%)	5 (12%)	.08
Nasal cannula ≤3 L/min	8	3 (5%)	5 (12%)	
Nasal cannula >3 L/min	5	5 (8%)	0	
Renal failure		9 (15%)	9 (21%)	.44
Grade 1	10	4 (7%)	6 (14%)	
Grade 2	8	5 (8%)	3 (7%)	
Liver failure		25 (42%)	16 (38%)	.82
Grade 2	25	16 (27%)	9 (21%)	
≥ Grade 3	16	9 (15%)	7 (17%)	
Altered mental status	15	12 (20%)	3 (7%)	.09

Values are bold when *P* is statisctically significant (*P* < .05).

Grading was assessed according to CTCAE v4.0 grading.

Abbreviations: BM, bone marrow; CRS, cytokine release syndrome; Haplo‐SCT, haploidentical stem cell transplantation; PBSC, peripheral blood stem cell.

### 3.2 Outcomes after Haplo‐SCT and CRS

One‐year OS and NRM rates for the whole population were 68% (95% CI: 58‐77) and 19% (95% CI: 12‐27), respectively. Six‐month cumulative incidence of grade 2‐4 aGVHD and 2‐year moderate‐severe cGVHD were 29% (95% CI: 20‐38) and 8.5% (95% CI: 4‐15), respectively.

**Figure 2:**
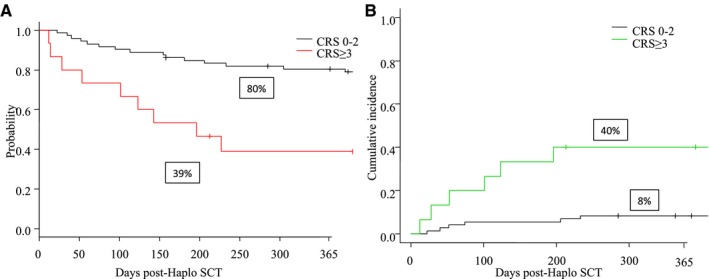
A, One‐year OS for CRS ≥3 vs <3 after Haplo‐SCT with PT‐Cy. B, One‐year NRM for CRS ≥3 vs <3 after Haplo‐SCT with PT‐Cy. CRS, cytokine release syndrome; Haplo‐SCT, haploidentical stem cell transplantation; NRM, nonrelapse mortality; OS, overall survival; PT‐Cy, posttransplant cyclophosphamide

While OS and NRM did not differ between patients experiencing grade ≥2 CRS vs grade <2 (data not shown), the outcome of recipients with grade ≥3 CRS was significantly worse compared with grade <3 CRS: 1‐year OS was 39% (95% CI: 15‐62) vs 80% (95% CI: 69‐88) (*P* = .002, Figure 2A), 1‐year NRM was 40% (95% CI: 15‐64) vs 8% (95% CI: 3‐16) (*P* = .005, Figure 2B).

By univariate analysis, the other variables affecting OS and NRM were represented by pretransplant disease status (Table 4) and recipient age (1‐year OS, hazard ratio [HR]: 1.02, *P* = .02; 1‐year NRM, HR: 1.03, *P* = .04, data not shown). Of note, NRM was much higher after PBSC relative to a BM graft, but this difference did not reach a statistical significance (20% vs 3%, *P* = .07).

**Table 4:** Univariate analysis of the outcome of patients receiving PBSC Haplo‐SCT for CRS ≥3, NRM and OS

Characteristics	CI CRS 3‐4	*P*	1y‐NRM	*P*	1 y‐OS	*P*
Disease type		.59		.16		.09
Myeloid	17% (7‐30)		17% (7‐31)		65% (47‐79)	
Lymphoid	13% (6‐23)		12% (5‐22)		79% (65‐88)	
Disease status pre‐Allo		**.002**		.64		**.0002**
CR	8% (3‐16)		9% (3‐19)		85% (73‐92)	
PR	11% (2‐30)		25% (7‐48)		63% (35‐81)	
SD/PD	38% (18‐58)		18% (4‐39)		45% (21‐67)	
Conditioning		.99		.76		.44
Nonmyeloblative	14% (5‐28)		16% (6‐31)		74% (55‐86)	
RIC	15% (7‐26)		14% (6‐27)		69% (52‐81)	
MAC	14% (2‐38)		10% (0‐37)		80% (41‐95)	
HCT‐CI		.39		.17		.65
0‐2	11% (5‐22)		7% (2‐17)		73% (58‐84)	
>3	17% (8‐29)		22% (11‐36)		75% (59‐86)	
CMV serostatus		.67		.71		.09
Neg→Pos	12% (2‐32)		14% (2‐38)		86% (54‐96)	
Others	16% (9‐24)		14% (0‐23)		71% (59‐80)	
Gender D/R		.48		.97		.79
Female→male	19% (6‐38)		13% (2‐34)		68% (40‐85)	
Others	14% (7‐22)		14% (7‐23)		75% (63‐83)	
Graft source		.07		.07		.63
BM	7% (2‐18)		3% (0‐14)		78% (59‐89)	
PBSC	20% (11‐31)		20% (11‐32)		71% (57‐81)	
HLA		.31		.67		.67
No	12% (6‐21)		76% (63‐85)		76% (63‐85)	
GVHD	21% (8‐37)		67% (44‐82)		67% (44‐82)	
HLA		.34		.65		.64
No	19% (9‐27)		73% (60‐83)		73% (60‐83)	
HVG	9% (2‐23)		74% (53‐87)		74% (53‐87)	
HLA‐GVHD		**.007**		.72		.83
No DRB1	14% (6‐21)		74% (62‐82)		14% (7‐22)	
DRB1 GVHD	57% (0‐82)		71% (26‐92)		14% (0‐50)	
CRS		NA		**.005**		**.002**
0‐2	NA		8% (3‐16)		80% (69‐88)	
≥3			40% (15‐64)		39% (15‐62)	

Values are bold when *P* is statisctically significant (*P* < .05).

Abbreviations: BM, bone marrow; CI, cumulative incidence; CMV, cytomegalovirus; CR, complete remission; CRS, cytokine releasing syndrome; GVHD, graft‐vs‐host‐disease; Haplo‐SCT, haploidentical stem cell transplantation; HCT‐CI, hematopoietic cell transplant‐comorbidity index; MAC, myeloablative conditioning; NRM, nonrelapse mortality; OS, overall survival; PBSC, peripheral blood stem cell; PD, progressive disease; PR, partial remission; RIC, reduced intensity conditioning; SD, stable disease.

Day 30 neutrophil engraftment did not differ between CRS ≥3 vs 2 vs patients with no CRS: 97% vs 100% vs 100% (*P* = .86). Grade 2‐4 aGVHD occurred more frequently among patients with grade 2 and grade ≥3 CRS relative to no CRS (36% vs 33% vs 14%), but the difference was not statistically significant (*P* = .38). Cumulative incidence of moderate‐severe cGVHD was higher after grade 2 or ≥3 CRS relative to no CRS (7% vs 13% vs 0%), but did not reach statistical significance (*P* = .37).

### 3.3 Risks factors for CRS

Because severe CRS ≥3 was the only type of CRS affecting the final outcome, we restricted our analysis only to identify risk factors for severe CRS. By univariate analysis, variables associated with increased incidence of grade ≥3 CRS were (Table 4): pretransplant disease status (38% for patients in stable [SD] or progressive disease [PD] vs 11% for those in partial remission vs 8% for those in complete remission [CR]; *P* = .002), graft source (PBSC vs BM: 20% vs 7%, *P* = .07), and HLA class II DRB1 mismatching in the GVHD direction (57% vs 14%, *P* = .007). Of note, neither recipient or donor age (data not shown), nor HLA mismatching in the GVHD direction on class I and other class II loci or CD34 cell dose were predictive risk factors for severe CRS (Table 4). By multivariable analysis (Table S2), active pretransplant disease (SD/PD) relative to CR status and HLA‐DRB1 mismatching in the GVHD direction remained independent predictors for increased risk of grade ≥3 CRS (HR: 14.3, *P* = .001, and HR: 17.2, *P* = .003, respectively).

## 4 DISCUSSION

In this report we have confirmed that grade ≥3 CRS is associated with a worse outcome in patients receiving Haplo‐SCT with PT‐Cy both in terms of OS and NRM. Our results extends previous observations on risk factors for the development of life‐threatening CRS since we have identified that disease burden (pretransplant active disease), HLA‐DRB1 mismatching, and graft type (PBSC vs BM) were significantly associated with a higher incidence of grade ≥3 CRS.

CRS has been described as a life‐threatening side effect not only after CAR‐T cells or bispecific antibodies^5^, ^6^ but recently also after Haplo‐SCT with PT‐Cy as GVHD prophylaxis.^7^ A similar finding was reported by Raj et al^8^ that did not find a statistically significant association between NRM and grade ≥2 CRS, but a tendency of greater NRM for grade ≥3 CRS by multivariable analysis. Consistent with these observations, we have found that grade ≥3 CRS, but not grade 2 CRS, was associated with worse OS (39% vs 80%, *P* = .002) and NRM (40% vs 8%, *P* = .005; Figure 2). Similar to Abboud et al,^7^ only one death in our cohort was directly attributable to CRS, while the increased death rate for patients with grade ≥3 CRS was due to other causes not directly correlated with CRS. This implies, as observed by Abboud et al,^7^ that CRS in the immediate posttransplantation setting may have long‐term side effects outside the time window of CRS occurrence (ie, day 0‐14). Similar to Abboud,^7^ we have identified a tendency for an increased incidence of aGVHD after grade 2 or ≥3 CRS, but GVHD alone does not explain alone the higher NRM rate within this cohort. This may be due to other mechanisms related to the cytokine storm, such as endothelial damage or macrophage activation. Unfortunately, we were not able to quantify cytokine storm and markers of endothelial damage due to the retrospective nature of our study. It is important to note that also patients receiving a PBSC graft were at higher risk of NRM relative to patients transplanted with a BM graft, but this difference did not reach statistical significance probably due to our small sample size. This may be due to the higher incidence of grade ≥3 CRS or other complication, such as GVHD as recently reported by retrospective studies.^24^, ^25^

The most common manifestation of CRS is represented by a noninfectious fever, that was originally named IFRT, occurring in the very first days after T‐cell‐replete Haplo‐SCT with PT‐Cy in approximately 30%‐90%^11^, ^12^, ^13^ of the cases and usually resolving 24 hours after the last dose of cyclophosphamide without any steroid treatment.^14^ More recently, McCurdy et al^15^ reported an incidence of early fever of 53% after Haplo‐SCT using BM as graft source. The authors reported a very low NRM rate, ranging between 8% and 14% depending on the intensity of the conditioning regimen. Cumulative incidence of grade 1 CRS was higher in our study because most of our patients received PBSC instead of BM as graft source and also because CRS was followed up to day 14 as in Abboud^7^ report, instead of day 6 as in the study by McCurdy et al.^15^ When we considered grade 1 CRS occurring within day 6 post‐SCT, 57% of the patients transplanted with BM (data not shown) experienced early fever similar to McCurdy et al.^15^ Consistent with the findings from the John Hopkins group, grade 1 CRS was not significantly associated with different OS (73% vs 71%, *P* = .69) and NRM (16% vs 14%, *P* = .71).

Frequency of grade ≥3 CRS was similar in our cohort (15%) to previous findings by Abboud et al (12%)^7^ and Raj et al (11%).^8^ Our study not only confirms, but extends previous reports on risk factors contributing to the occurrence of severe CRS. While the study from the Washington University of St Louis^7^ did not identify any risk factors, Raj et al^8^ demonstrated a higher incidence and severity of CRS after PBSC relative to BM cells. In this study we confirmed these observations, since recipients of PBSC grafts were at higher risk of developing high fever for grade 1 CRS (*P* = .0005). Moreover, grade ≥3 CRS was three times more frequent after PBSC relative to a BM graft (*P* = .07). This observation may suggest that T or CD34^+^ cell doses are central mediators of CRS, but neither Abboud^7^ nor Raj^8^ were able to correlate the severity of CRS with T or CD34^+^ contents of the graft. In marked contrast, McCurdy et al^15^ identified a strong correlation between early fever and CD3^+^ cell content. Unfortunately, we were not able to correlate the incidence of CRS with CD3^+^ cell content of the graft due to missing data. In any case, we did not see any association between CD34^+^ cell dose and grade ≥3 CRS. This suggests the importance of other factors that may mediate CRS, such as HLA‐DRB1 mismatching and tumor burden that we were able to identify for the first time as variables associated with grade ≥3 CRS in the contest of Haplo‐SCT with PT‐Cy. McCurdy et al^15^ reported for the first time a correlation between class II HLA mismatching and early fever after Haplo‐SCT. In agreement with this finding we observed that HLA mismatching at the DRB1 locus in the GVHD direction was associated with higher incidence of grade ≥3 CRS, suggesting an important role for CD4^+^ cells and class II HLA‐mediated presentation in the pathophysiology of CRS. Unfortunately, data relative to HLA DPB1 mismatching were not available in our study. Another interesting finding of this report is the increased incidence of grade ≥3 CRS for patients with high tumor burden. This observation is an agreement with several reports showing that disease burden is among the most important predictors of severe CRS after CAR‐T cell therapy or bispecific T‐cell engager administration.^26^, ^27^, ^28^, ^29^ All together these observations may help identify patients at higher risk for life‐threatening CRS and develop models for prevention (such as BM graft for patients with active disease status or class II HLA mismatch) or early intervention (for instance, with anti‐IL6 antibody) at the first signs of CRS in patients at higher risk of CRS. In this contest, tocilizumab has recently emerged has a promising therapy for patients with CRS after Haplo‐SCT.^30^, ^31^ In our study, tocilizumab was employed only in one patient who eventually died of multiorgan failure on day 28 post‐SCT.

It is important to acknowledge the limitations of this study due to its retrospective nature, the limited number of patients, and the single‐center setting. The next step of our study is to participate to a multi‐center collaboration in order to confirm and extend our preliminary findings. This effort can be of translational relevance in order to develop a clinical trial for prevention or preemptive treatment of life‐threatening CRS in a cohort of patients at high risk to develop such complication. Deepening our understanding of CRS biology, by analyzing not only the T cell, but also the macrophage^32^ and endothelial compartment, is warranted in order to improve our chances to predict and abrogate CRS after T‐cell‐replete Haplo‐SCT.

In summary, our analyses illustrate that grade ≥3 CRS is a life‐threatening side effect after Haplo‐SCT with PT‐Cy and that high tumor burden, class II HLA mismatching and graft source are important predictors of such complication. Therefore, selection of the best available donor, optimization of pretransplant tumor response and selection of the most appropriate graft source are all important factors to take into consideration to optimize the outcome of patients receiving Haplo‐SCT with PT‐Cy.

## CONFLICT OF INTEREST

The authors have no conflict of interest to disclose.

## AUTHOR CONTRIBUTION

JM collected and analyzed data, and wrote the manuscript. DT and FM collected data. SB, BS, LM, and CDP contributed to data collection. CDV and DM provided intellectual contribution. CC‐S, MDP, and AS reviewed the manuscript. LC supervised the project, discussed data analysis, and reviewed the manuscript.

## Data Availability

The data that support the findings of this study are available on request from the corresponding author. The data are not publicly available due to privacy or ethical restrictions.
