# Supplementary material for: Pretransplant active disease status and HLA class II mismatching are associated with increased incidence and severity of cytokine release syndrome after haploidentical transplantation with posttransplant cyclophosphamide
Source: Cancer Med. 2019 Nov 8;9(1):52–61. doi: 10.1002/cam4.2607 (PMC6943086; doi:10.1002/cam4.2607)
Supplement: Supplementary file 1 [file CAM4-9-52-s001.doc]

**Supplementary Table 1.** Grading of Cytokine Release Syndrome, modified from Lee et al and Abboud et al

| **Grade 1** | **Symptoms are not life threatening and require symptomatic treatment only, eg, fever, nausea, fatigue, headache, myalgias, malaise** |
| --- | --- |
| **Grade 2** | Symptoms require and respond to moderate intervention:  - Oxygen requirement < 40%, ≤ 3 L nasal cannula or  - Hypotension responsive to fluids or low dose of one vasopressor or  - Grade 2 organ toxicity |
| **Grade 3** | Symptoms require and respond to aggressive intervention:  - Oxygen requirement ≥ 40%, >3 L nasal cannula or  - Hypotension requiring high dose or multiple vasopressors or  - Grade 3 organ toxicity or grade 4 transaminitis  New-onset altered mental status without other explanation*  New cardiomyopathy without wall motion abnormality |
| **Grade 4** | Life-threatening symptoms:  - Requirement for ventilator support or  - Grade 4 organ toxicity (excluding transaminitis) |
| **Grade 5** | Death |

Table Legend: Grades 2-4 refer to CTCAE v4.0 grading.* Altered mental status of

sufficient severity to warrant investigation by head imaging and or lumbar puncture.
