# Supplementary material for: Pretransplant active disease status and HLA class II mismatching are associated with increased incidence and severity of cytokine release syndrome after haploidentical transplantation with posttransplant cyclophosphamide
Source: Cancer Med. 2019 Nov 8;9(1):52–61. doi: 10.1002/cam4.2607 (PMC6943086; doi:10.1002/cam4.2607)
Supplement: Supplementary file 2 [file CAM4-9-52-s002.docx]

Supplementary Table II. Multivariate analysis of variables affecting probability of CRS 3-4

| **Characteristics** | **CRS 3-4** | **p** |
| --- | --- | --- |
| **Disease status pre-Allo**  CR  PR  SD/PD | 1  1.81 (0.26-7.12.64)  14.33 (3.07-66.88) | 0.54  **0.001** |
| **HLA**  **No DRB1**  **DRB1-GVHD** | 0  17.19 (2.61-113.02) | **0.003** |
